# Calibration and count rate performance of NaI(Tl) gamma counters for quantifying the activity ^89^Zr radiopharmaceuticals

**DOI:** 10.1002/acm2.70788

**Published:** 2026-09-18

**Authors:** Michael Silosky

**Affiliations:** ^1^ Department of Radiology University of Colorado Anschutz Medical Campus Aurora Colorado USA

**Keywords:** ^89^Zr, calibration, gamma counters

## Abstract

**Background:**

Quantification of low activity samples using gamma counters is a common technique when investigating the biodistribution of experimental radiopharmaceuticals. Several human and pre‐clinical trials have investigated ^89^Zr labeled compounds, utilizing gamma counter to quantify activity in blood or animal samples. While the performance of gamma counters has been investigated for other positron emitting radionuclides, the more complex spectrum, including a 909 keV gamma emission, and low positron fraction of ^89^Zr may reduce both counting efficiency while increasing the severity of count rate losses.

**Purpose:**

The purpose of this study is to evaluate both the counting efficiency and the effect of count rate losses on the quantitation of activity for ^89^Zr using NaI(Tl) gamma counters.

**Methods:**

Sources of known activity (initially 78.3 kBq) were measured in a gamma counter as they decayed over approximately 15 days. Observed count rates were fit with a paralyzable detector model for two separate energy windows, 511 keV ± 10% and 909 keV ± 10%, as well as their sum. Counting efficiency in the absence of losses as well as the effective deadtime for each dataset were calculated.

**Results:**

Count rates for each energy window were well fit with a paralyzable model with *R* ^2^ > 0.999. Counting efficiency (mean ± 95% confidence interval) was 10.07% ± 0.07%, 17.38% ± 0.13%, and 27.46% ± 0.16% for the 511 keV, 909 keV, and summed energy windows respectively. Effective dead time (mean, 95% confidence interval) was 14.31 µs, (13.30 µs–15.30 µs), 7.11 µs (6.68 µs–7.55 µs), and 4.77 µs (4.48 µs–5.06 µs) for the 511 keV, 909 keV, and summed energy windows respectively.

**Conclusion:**

Counting efficiency and count rate performance for ^89^Zr are poorer than for other positron emitting radionuclides. Count rate losses follow the paralyzable model and are correctible.

## 1 INTRODUCTION

Zirconium‐89 (^89^Zr) is a positron‐emitting radionuclide that has been identified as well‐suited for the labeling and imaging of monoclonal antibodies.^1^ While ^89^Zr radiopharmaceuticals are not yet used in a clinical capacity, there have been a number of preclinical and human trials evaluating the safety and efficacy of ^89^Zr labelled compounds as an immuno‐positron emission tomography (PET) imaging agent.^2^ Evaluation of the pharmacokinetic characteristics of these agents typically involves the measurement of activity within blood samples in human trials as well as organ and tissue samples in preclinical trials. In both cases, samples are measured in a NaI(Tl) gamma counter with count rates being converted to activity by applying pre‐determined efficiency factors. Of particular concern for preclinical trials is the relatively high specific activities that may be observed in certain samples. For example, a 2020 study of ^89^Zr zirconium‐p‐isothiocyanatobenzyl‐desferrioxamine included an administration of approximately 0.4 MBq (10.8 µCi) to mice. Forty‐eight hours post‐administration, multiple organs were found to have relatively high concentrations of the radiopharmaceutical, with the heart and lungs concentrating as much as 50% and nearly 40% of the remaining activity respectively in some mice.^3^ Given the 78.4 h half‐life of ^89^Zr, even at a lower concentration of 20% observed in other organs, activities would be approximately 0.052 MBq (1.4 µCi) after 48 h of decay.

**FIGURE 1:**
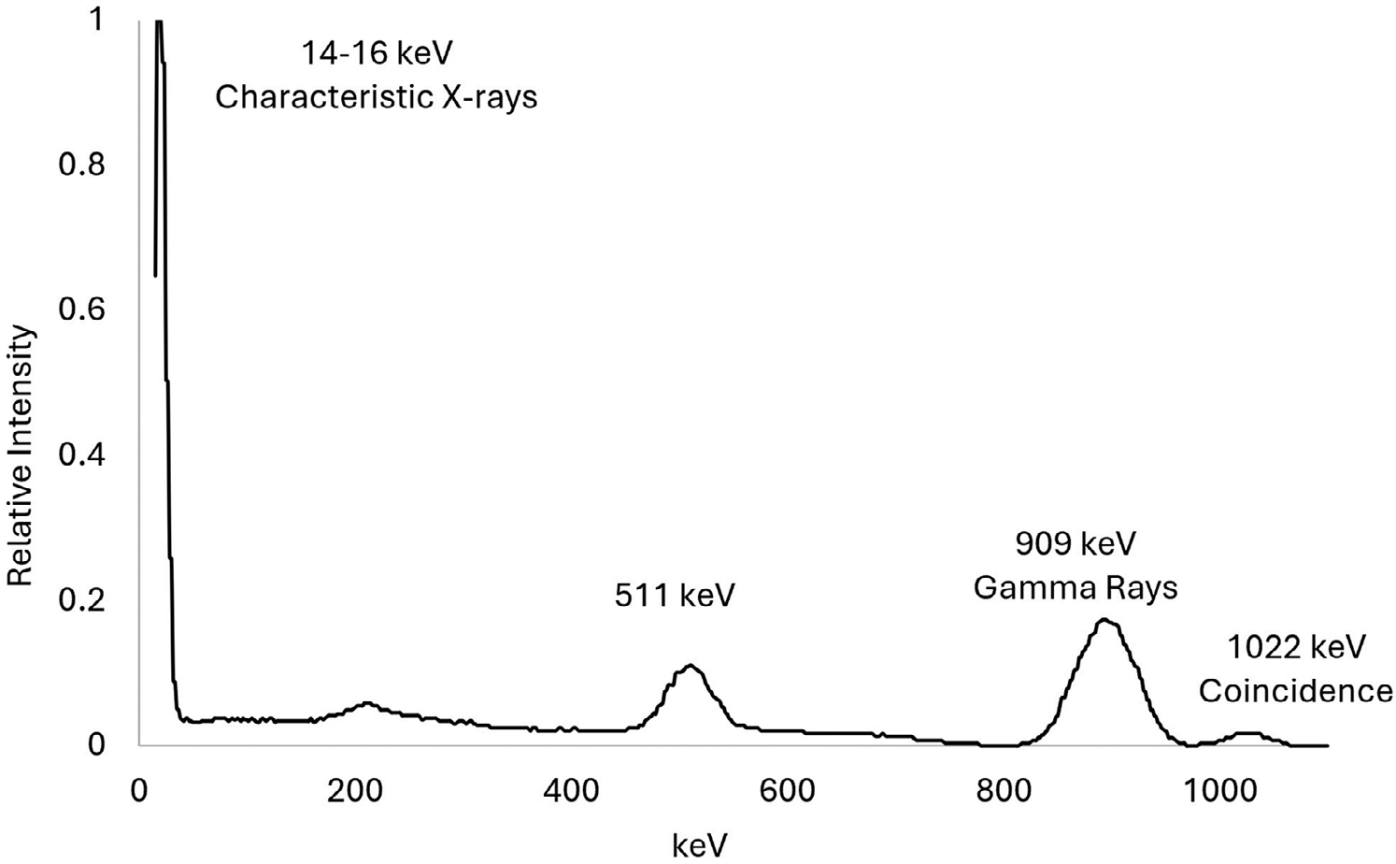
^89^Zr spectrum, showing the 511 keV positron peak and the 1022 keV coincidence peak. Also shown are the photopeaks for low‐energy characteristic x‐rays (14–16 keV) and 909 keV gamma rays.

The performance of NaI(Tl) gamma counters in the accurate quantification of activity has been well investigated for other positron‐emitting radionuclides, with 5% count rate losses observed at 0.063 MBq (1.7 µCi) for ^18^F.^4^ Because count rate is used to determine the activity of a sample, uncorrected losses directly cause an underestimation of activity. Further, these losses may be affected by the decay characteristics of the radionuclide. ^89^Zr decays to ^89m^Y via positron emission 22.3% of the time and via electron capture 76.6% of the time. From here, ^89m^Y decays to stable ^89^Y with a half‐life of 15.7 seconds, emitting a 909‐keV gamma ray. Figure 1 illustrates the spectral response of a NaI(Tl) gamma counter when measuring approximately 37 kBq (1 µCi) of ^89^Zr. While the ^18^F spectrum observed by Lodge et al. demonstrated a dominant peak at 511 keV and a smaller coincidence summing peak at 1022 keV, with no additional photopeaks,^4^ the ^89^Zr spectrum is more complex, with a gamma ray photopeak at 909 keV, a smaller peak at 511 keV due to the low positron emission fraction, a coincidence summing peak at 1022 keV, and a substantial low energy peak at 14–16 keV resulting from characteristic x‐rays produced as a result of electron capture.^5^ In other NaI(Tl) counting systems such as gamma cameras, count rate losses have been found to be dependent on the detectors spectral response, with the measured deadtime (τ) related to the ratio of counts within the energy window to total counts across the spectrum.^6^ Consequently, the spectral differences between ^89^Zr and ^18^F may cause count rate losses for ^89^Zr to be more significant than for ^18^F. The purpose of this study is to evaluate both the counting efficiency and the effect of count rate losses on the quantitation of activity for ^89^Zr using a NaI(Tl) gamma counter.

## 2 METHODS

To evaluate counting efficiency and characterize count rate losses, sources of known activity were prepared and periodically measured in a well counter as they decayed over a period of approximately 15 days. Observed count rates and activity were fit with a modified paralyzable detector model. To create the sources, 19.72 MBq (532.9 µCi) of ^89^Zr oxalate was diluted with water in a 250 mL volumetric flask with a tolerance of ± 0.12 mL. The diluted activity was determined by measuring a syringe both pre‐ and post‐dilution using a Capintec CRC 55TW dose calibrator (Mirion Technologies Capintec, Florham Park, NJ, USA) that had been cross calibrated to match the activity of a ^89^Zr source provided by the radionuclide manufacturer (Institute for Medical Research Cyclotron Laboratory, Madision WI, USA). A calibration setting had been identified which matched the nominal activity provided by the manufacturer for the dose calibrator of 34.74 MBq (0.94 mCi). Following a mixing procedure to ensure a uniform distribution, three 1 mL samples were pipetted into counting tubes made of 1‐mm‐thick clear plastic with a maximum capacity of 3 mL each. The micropipette used for this purpose had a manufacturer specified accuracy of ± 0.8%–1.8%. The nominal activity of the sources at the time of preparation was 79 kBq (2.13 µCi). A fourth tube was prepared with 1 mL of non‐radioactive water to be used for background correction during the counting procedure.

All counting was performed using a commercial well‐type gamma counter with a single NaI(Tl) detector (2480 Wizard,^2^ PerkinElmer, Waltham, MA, USA). Each sample along with the non‐radioactive water tube were counted for one minute. Two energy windows were used, one centered at 511 keV ± 10%and another centered at 909 keV ± 10%. These energy windows were chosen to allow direct comparison of the 511 and 909 keV energy windows without unintentionally including the signal from the coincidence summing peak. The procedure was repeated 8 times at periodic intervals until the sources had decayed to approximately 33 kBq (0.089 µCi). Repeat measurements were made at approximately 1, 2, 3, 5, 7, 9, 12, and 15 days post the initial measurement.

In the absence of count rate losses, several factors impact the counting efficiency of NaI(Tl) gamma counters, including the energy and relative frequency of emission, counting window, and the source and detector geometry. However, for the purpose of measuring a known radionuclide in a reproducible geometry, these factors may be represented in a single efficiency factor, S, specific to the instrument, radionuclide, and counting window. Here, S is the ratio of the count rate in the absence of count rate losses, n (hereafter referred to as the input count rate), to the source activity, A, such that

(1) $$S=\frac{n}{A}$$

Assuming a paralyzable detector, the observed count rate, m, is related to the input count rate, n, as

(2) $$m=ne^{-\left(n\tau \right)}$$

where τ represents the effective deadtime for the counting system for the specific spectral conditions and energy window being used.^6^ Substituting the product of S and A for the input count rate, the observed count rate is related to sample activity as

(3) $$m=\left(S\times A\right)e^{-\left(S\times A\times \tau \right)}$$

Both the 511 and 909 keV energy window count rates were modelled as function of activity with fit parameters S and τ using weighted least squares regression with 1/m weighting. All modeling was performed using GraphPad Prism version 10.1.1 (GraphPad Software, Boston, Massachusetts, USA), which provided the fit parameters and confidence intervals. The count rates from each sample were background corrected and input into the software with its own counting time as opposed to decay correcting to an initial time point and averaging. Additionally, the count rates for both energy windows were summed at each time point and also modelled using this method. The percent count rate loss at each activity was also calculated.

### 2.1 Results

**FIGURE 2:**
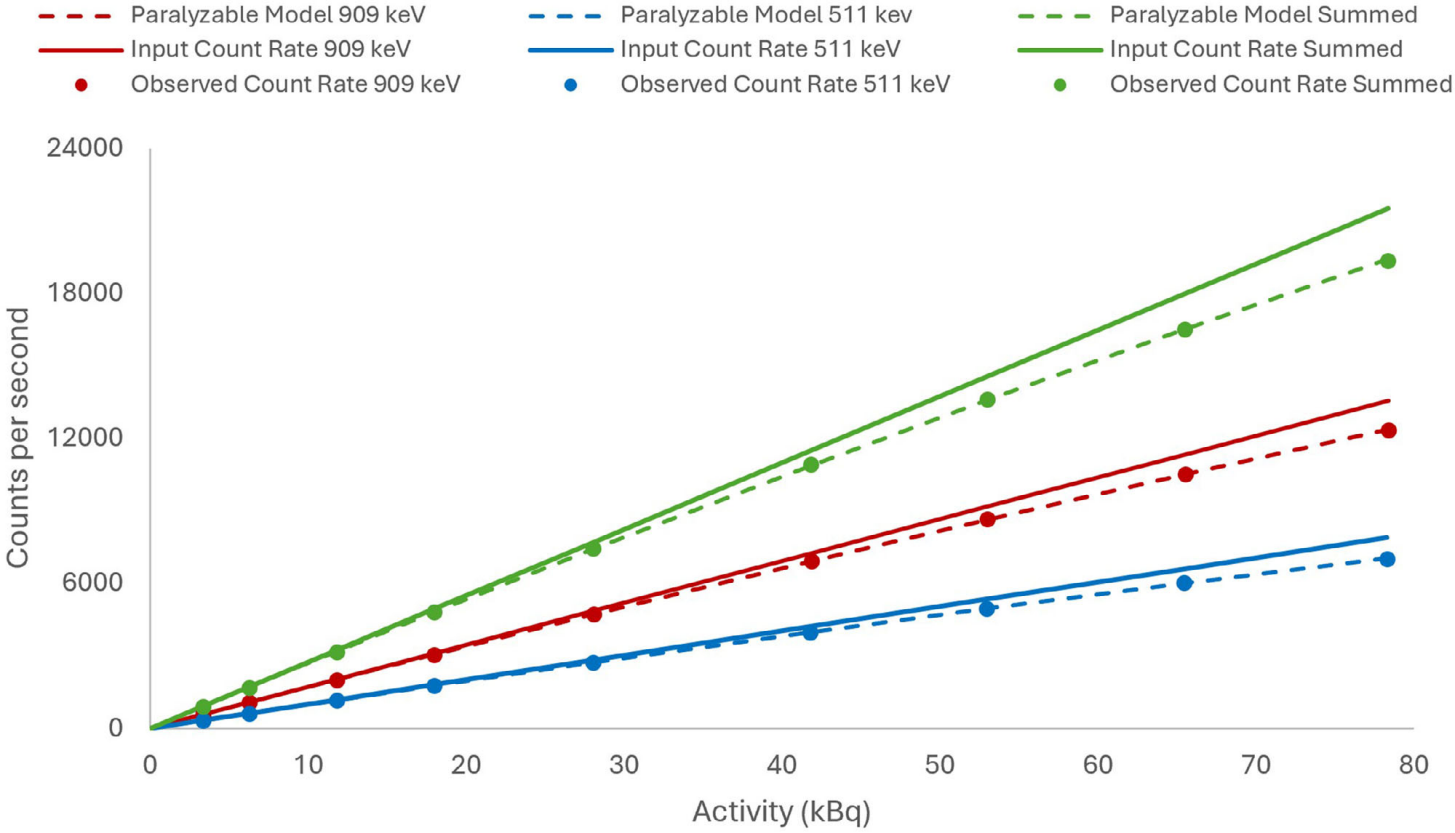
Observed, input, and modelled count rate for the 909 keV, 511 keV, and summed energy windows demonstrating *R* ^2^ > 0.999 agreement between the observed count rates and paralyzable model. Data points represent the average count rates and activities of the 3 sources at each measurement time point.

For both the 511 and 909 keV energy windows, as well as the summed data, the observed count rate was well modelled as a function of activity using Equation 3, where *R* ^2^ > 0.999 in each case. Table 1 shows the fit parameters and their associated 95% confidence intervals as well as the percent loss at select activity levels. Figure 2 shows plots of the observed count rates, the paralyzable model fit to each data set, and the theoretical input count rates in the absence of count rate losses. Even at a modest activity of 37 kBq (1 µCi), losses were found to be between 4.47%–5.19% depending on the energy window selected.

**TABLE 1:** Fit parameters and confidence intervals for Equation 2 as well as percent count rate losses for the 511 keV, 909 keV, and summed energy windows.

	*S*		*τ* (µs)		% Count Rate Loss			
	Fit	95% CI	Fit	95% CI	22 kBq	37 kBq	63 kBq	93 kBq
**511** **keV**	0.1007	0.1000–0.1014	14.31	13.30–15.30	3.12	5.19	8.68	12.54
**909** **keV**	0.1738	0.1730–0.1746	7.11	6.68–7.55	2.68	4.47	7.49	10.86
**Summed**	0.2746	0.2733–0.2759	4.77	4.48–5.06	2.84	4.73	7.92	11.47

The coefficient of variation in counts per second at each datapoint was less than 1%. Consequently, error bars have been omitted as they cannot be visualized given the scale of the individual data point indicators in the plot.

## 3 DISCUSSION

As mentioned, similar work has been performed evaluating the efficiency and count rate performance for other positron‐emitting radionuclides. For ^18^F, Lodge et al. noted count rate losses of 1% and 5% at activities of 22 kBq and 63 kBq, respectively.^4^ As shown in Table 1, losses for ^89^Zr were found to be 3.12% and 8.68% at these same activities, respectively, when using the same 511 keV energy window. Regarding efficiency, Lodge et al. observed 34.4% ± 0.18% and 30.1% ± 0.07% for ^18^F and ^68^Ga, respectively. They also reported these efficiencies after accounting for positron fraction as 35.5% ± 0.18% and 33.8% ± 0.07%, respectively, commenting that these values are in close agreement.^4^ For the 511 keV energy window, the efficiency for ^89^Zr was found to be 10.07% with a value of 45.16% when adjusting for positron fraction. While this is notably higher than what Lodge et al. reported for ^18^F and ^68^Ga, the difference is most likely the result of substantial down‐scatter from the 909 keV gamma rays that can be readily visualized in Figure 1.

Regarding the deadtime parameter, τ, the manufacturer's stated system deadtime is 2.5 µs. As previously observed,^6^ estimates of τ are dependent upon how much of the energy spectrum is captured within the applied energy window. As listed in Table 1, the estimated dead time for ^89^Zr was found to be 14.31 and 7.11 µs in the 511 and 909 keV energy windows, respectively. The estimate of τ drops to 4.77 µs when the 511 and 909 keV windows are summed. This appears reasonable given the amount of signal outside these windows, approximately 62% of the total signal, particularly from low‐energy characteristic x‐rays and scatter.

Based on these observations, it is recommended that if a single energy window is used, it should be centered around the 909 keV photopeak for its increased signal relative to the 511 keV photopeak. That being said, summing two energy windows that capture both the 511 keV peak and the 909 keV peak individually will result in the greatest efficiency. Regardless of the energy window selected, count rate losses can be substantial for ^89^Zr. For accurate quantitation of sample activity, it is recommended that samples either be allowed to decay to relatively low values before measurement (below 7 kBq to reduce losses to less than 1%) or that correction factors adjusting for count rate losses are applied. Table 2 provides an example of how such corrections may be used.

**TABLE 2:** Correction factors for count rate losses have been provided for a range of observed count rates using the 909 keV energy window data as an example.

909 keV (*S* = 0.1738, *τ* = 7.11 µs)			
Observed Count Rate (CPS)	Correction Factor	Input Count Rate (DPS)	Activity (kBq)
15000	1.128	16917	97.336
12500	1.103	13787	79.327
10000	1.080	10798	62.129
7500	1.058	7935	45.656
5000	1.038	5188	29.850
2500	1.018	2546	14.649

The absolute quantification and precision of activity using these methods is directly dependent on accuracy of the activity of the prepared sources. While this includes pipetting error, that value is relatively small, between 0.8% and 1.8% as stated by the manufacturer. However, it also includes error in the dose calibrator measured activity used for dilution. If the dose calibrator setting is incorrect, then the diluted activity will be incorrect, and the accuracy of the well counter efficiency will suffer. This presents a challenge as traceable standards for ^89^Zr are not available and facilities are dependent on calibration through comparison to the manufacturer's stated activity, with its uncertainty. It also makes it quite challenging to fully quantify the error in the entire process. However, for the purpose of determining relative uptake, as in preclinical studies using mice, an inaccuracy in the administered activity measured using the dose calibrator and an inaccuracy in the well counter efficiency would cancel out mathematically, and percentage uptake in particular organs would remain unaffected.

It should be noted that, while the fit parameters and correction factors are specific to the manufacturer and model of the instrument used in this study, these methods are likely to be generalizable, not only to other well counters, but also other radionuclides, assuming the devices follow the paralyzable detector model. The applicability of these methods for other well counter models should be evaluated, ensuring that count rate performance behaves appropriately. For studies requiring the quantification of activity of samples that may suffer from count rate losses, consideration should be given to evaluating these effects in advance and providing a means to correct for them.

## 4 CONCLUSION

This work assessed the counting efficiency and count rate performance of a NaI(Tl) gamma counter for the measurement of ^89^Zr radiopharmaceuticals. As expected, both efficiency and count rate performance were poorer than what has been reported for other positron‐emitting radionuclides. Because count rate losses are demonstrated to follow the paralyzable detector model, losses at different source activities are predictable and may be corrected.

## AUTHOR CONTRIBUTIONS

Michael Silosky is the sole author of this work and was fully responsible for conception, experimental design, data collection, analysis, and writing of this manuscript.

## CONFLICT OF INTEREST STATEMENT

The author declares no conflicts of interest.

## Data Availability

Data used in this manuscript will be provided by the authors upon request.
